# Complete chloroplast genome and phylogenetic analysis of *Bupleurum kaoi* Liu, Chao, and Chuang, 1977: an endemic species in Taiwan

**DOI:** 10.1080/23802359.2022.2082892

**Published:** 2022-08-22

**Authors:** Chi-Chun Huang, Chao-Li Huang, Tsai-Wen Hsu, Li-Hui Chang, Kuo-Hsiang Hung, Wei-Kuang Wang

**Affiliations:** a Endemic Species Research Institute, Jiji, Nantou, Taiwan; b Institute of Tropical Plant Sciences and Microbiology, National Cheng Kung University, Tainan, Taiwan; c Graduate Institute of Bioresources, Pingtung University of Science and Technology, Pingtung, Taiwan; d Department of Environmental Engineering and Science, Feng Chia University, Taichung, Taiwan

**Keywords:** *Bupleurum kaoi*, chloroplast genome, phylogeny

## Abstract

*Bupleurum kaoi* Liu, Chao, and Chuang is an endemic and endangered herb in Taiwan. In this study, the complete circular chloroplast genome of *B. kaoi* was reconstructed and annotated using Illumina sequencing. The genome size of *B. kaoi* is 155,938 bp, including a pair of inverted repeat regions (IRs: 26308 bp), separated by a large single-copy (LSC) region of 85,784 bp and a small single-copy (SSC) region of 17,538 bp. The GC content of the chloroplast genome is 37.6%. There are 113 different genes in the chloroplast genome of *B. kaoi*, including 79 protein-coding genes, 30 tRNA genes, and four rRNA genes. A maximum-likelihood phylogenetic analysis showed that *Bupleurum* species is the monophyletic group, and *B. kaoi* belongs to subgenus *Bupleurum* and is closely related to *B. scorzonerifolium.*

*Bupleurum kaoi* Liu, Chao, and Chuang, 1977, an endemic plant in Taiwan, is a perennial herb in the Apiaceae family (Kao 1996). In Taiwan, *B. kaoi* is distributed at low altitudes in the northern and central regions. *Bupleurum* has great commercial value as a traditional medicine in China, Japan, and some other Asian countries (Yang et al. 2017). Its most effective metabolite, saikosaponins, is a triterpenoid that possesses immunomodulatory, hepatoprotective, and antitumor abilities (Chiang et al. 2003). Hu et al. (2016) reported that higher saikosaponin content and greater antimelanoma activity were detected in *B. kaoi* than in *Bupleurum chinense* DC, 1830, a traditional Chinese medicinal herb. However, *B. kaoi* is regarded as an endangered species because of human overexploitation and habitat destruction (Editorial Committee of the Red List of Taiwan Plants 2017). In the present study, the complete chloroplast genome of *B. kaoi* is presented based on next-generation sequencing. The chloroplast genome will contribute to understanding the phylogenetic relationship of *B*. *kaoi.*

A wild individual of *B. kaoi* was collected from Tongxiao township (120°42′00″E, 24°31′48″N), Miaoli County, Taiwan. *B. kaoi* is not a legally protected species in Taiwan, despite the decline of population size. The collection location in this study is not a privately-owned or protected area. No permits were required for this study. The voucher specimen (TAIE No. 47911) was deposited at the herbarium of the Endemic Species Research Institute (Chi-Chun Huang, cchuang@tesri.gov.tw). Total genomic DNA was extracted from the leaf materials of *B. kaoi* using CTAB extraction (Doyle and Doyle 1991). The library was sequenced by the Illumina NovaSeq 6000 platform with the double terminal sequencing method (pair-end 150). In total, 13.7 Gb of clean data was generated and used to assemble the chloroplast genome using MEGAHIT v1.0 (Li et al. 2016). Annotation of chloroplast genome was performed using GeSeq (Tillich et al. 2017).The annotated genomic sequence has been deposited in GenBank under accession number OK050523.

The structure of the chloroplast genome of *B. kaoi* was circular, and the size was 155,938 bp. It was composed of a pair of inverted repeat regions (IRs: 26,308 bp) separated by a large single-copy (LSC) region of 85,784 bp and a small single-copy (SSC) region of 17,538 bp. The GC content of the chloroplast genome is 37.6%. There are 113 different genes in the chloroplast genome of *B. kaoi*, including 79 protein-coding genes, 30 tRNA genes, and four rRNA genes. Furthermore, 14 (nine protein-coding and five tRNA genes) genes contain one intron and three protein-coding genes (*clpP1*, *pafI,* and *rps12*) contain two introns. *rps12* has been recognized as a trans-splicing gene.

The phylogenetic tree was reconstructed with the complete chloroplast genomes of *B. kaoi*, another 36 species in the Apiaceae family and *Panax ginseng* in the Araliaceae family. Sequence alignment was conducted using the MAFFT online server (Katoh et al. 2018), and subsequently, a maximum likelihood phylogenetic tree was reconstructed by MEGA version X with 1000 bootstrap replicates (Kumar et al. 2018). The results showed that all *Bupleurum* species formed a monophyletic clade (Figure 1), which was consistent with Huang et al. (2021) study. Neves and Watson (2004) proposed the subdivision of *Bupleurum* into subgenus *Penniervia* and *Bupleurum*. *Bupleurum kaoi* is clustered with subgenus *Bupleurum* species and closely related to *B. scorzonerifolium* willd, distributed in Russia, Korea, Japan, and North China (She and Watson 2005). *Bupleurum kaoi* is the only indigenous species of *Bupleurum* reported in Taiwan. Geographic isolation (Taiwan Strait) is known to contribute to divergent evolution, resulting in the monophyly of *B. kaoi*. To elucidate the intraspecific relationship of *Bupleurum*, more complete chloroplast genomes are needed. This study enriches the chloroplast genome database of *Bupleurum* and provides a scientific basis for the *Bupleurum* phylogeny.

**Figure 1.**
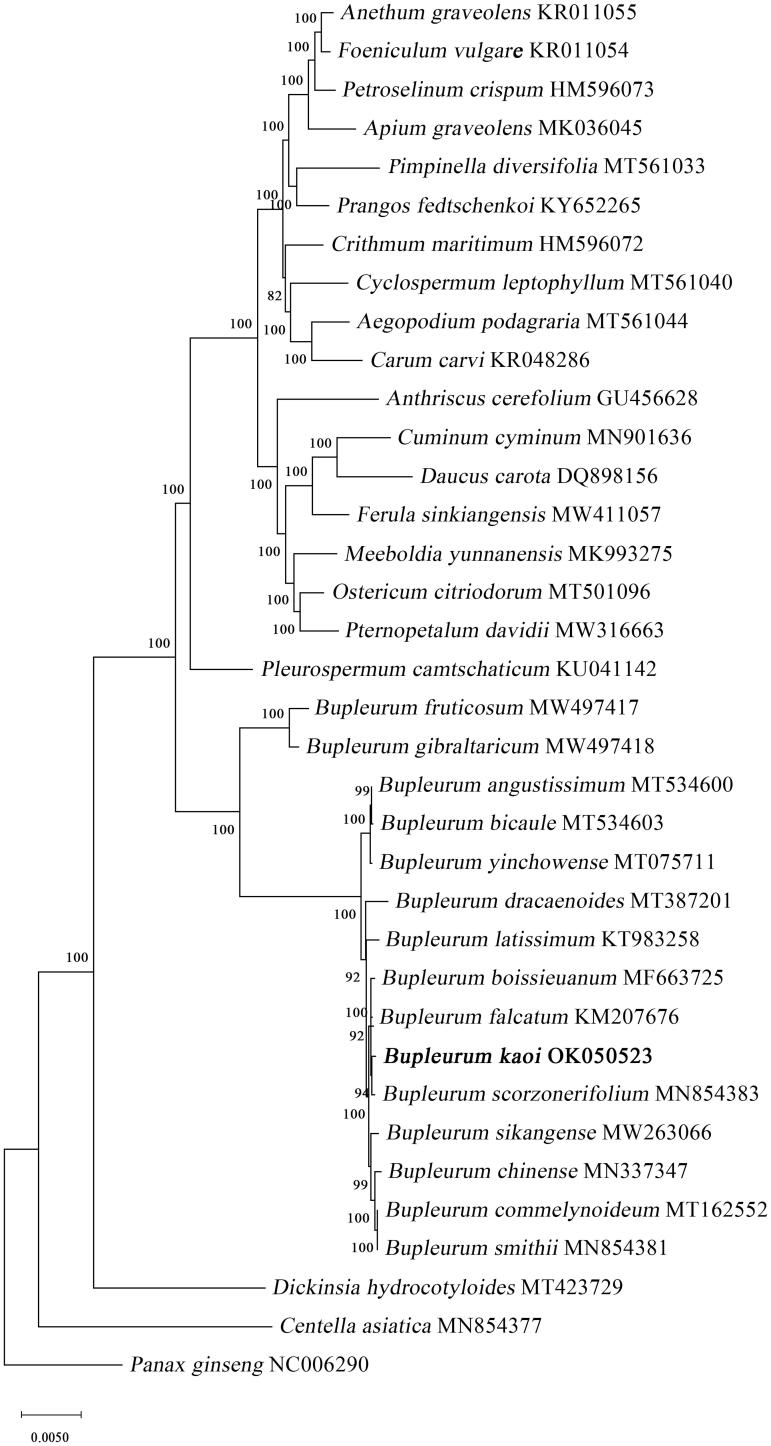
The phylogenetic tree was constructed using 36 chloroplast genome sequences based on the maximum-likelihood analysis. The numbers on the branches are bootstrap values.

## Authors’ contributions

Chi-Chun Huang, Chao-Li Huang, Kuo-Hsiang Hung, and Wei-Kuang Wang involved in the conception and design. Chi-Chun Huang, Tsai-Wen Hsu, and Li-Hui Chang involved in collection of materials. Chi-Chun Huang and Chao-Li Huang involved in analysis and interpretation of the data. Chi-Chun Huang, Chao-Li Huang, Kuo-Hsiang Hung, and Wei-Kuang Wang involved in the drafting of the paper. All authors agreed to be accountable for all aspects of this work.

## Data Availability

The genome sequence data that support the findings of this study are openly available in GenBank of NCBI at (https://www.ncbi.nlm.nih.gov/) under the accession no. OK050523. The associated BioProject, SRA, and Bio-Sample numbers are PRJNA766759, SRR16095050, and SAMN21876084, respectively.
